# Spontaneous Resolution of Paraparesis Because of Acute Spontaneous Thoracolumbar Epidural Hematoma

**Published:** 2012-01-01

**Authors:** M Gundag, M H Seyithanoglu, K Dogan, S Kitis, N Ozkan

**Affiliations:** 1 Department of Neurosurgery, Faculty of Medicine, Bezmialem Vakif University, Istanbul, Turkey; 2 Department of Neurosurgery, Ardahan State Hospital, Ardahan, Turkey; 3 Department of Neurosurgery, Faculty of Medicine, Abant Izzet Baysal University, Bolu, Turkey

**Keywords:** Spinal epidural hematoma, Cord compression, Conservative therapy

## Abstract

Symptomatic spontaneous spinal epidural hematoma(SSEH) is an uncommon cause of cord compression that commonly is considered as an indication for emergent surgical decompression. We aimed to investigate a patient with a SSEH that completely resolved clinically and radiographically, without surgical treatment. The patient presented three days after the sudden onset of back pain, numbness, and weakness. Magnetic Resonance Imaging (MRI) revealed a posterior thoracolumbar epidural hematoma extending from the level of T10 to L2 with significant cord compression. Decompression was recommended but he refused surgery and was managed conservatively. One month later, weakness totally recovered and hematoma was absent on MRI.

## Introduction

Spontaneous spinal epidural hematoma (SSEH) is an uncommon cause of cord compression and associated with vascular malformations, neoplasm, infections, coagulopathy, pregnancy and idiopathic causes.[1][2][3][4] Magnetic Resonance Imaging (MRI) is the gold stan­dard for diagnosis of SSEH. We want to indicate a pa­tient with a SSEH that a complete motor and sensory recovery was observed at 1-month follow up with reso­lution of the thoracolumbar epidural hematoma, clini­cally and radiographically, without surgical treatment.

## Case Report

**Fig. 1:**
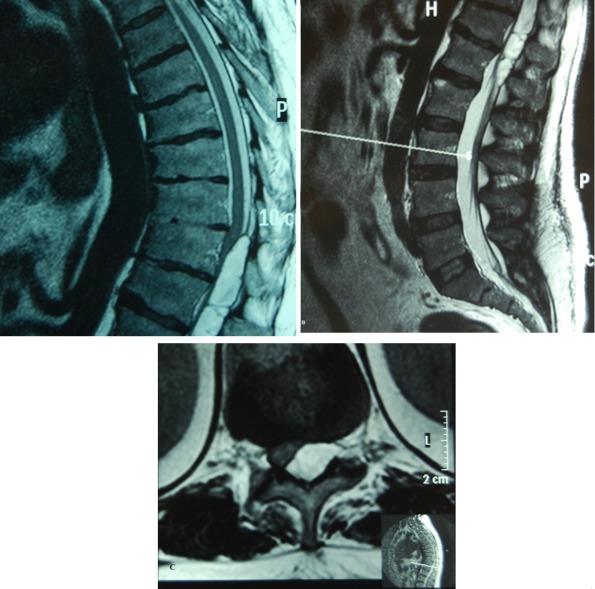
A, B) Sagittal T1W MRI images showing a well defined lesion in the posterior epidural space extending from T10 to L2 level, C) Axial T1W MRI image showing a hyperintense lesion in the posterior epidural space displacing the cord anteriorly and compressing it.

A 46-year old man presented 3 days after the sudden onset of back pain, numbness, and weakness of lower limbs after warfarin therapy for deep vein thrombosis. Clinical examination showed that the degree of motor weakness of both lower limbs was 3/5 and the level of numbness was T11 dermatome. Reflexes were depressed. Rectal examination showed normal anal tone and urinary retention was not detected. There was no neurological deficit in the upper limbs. The MRI revealed a posterior thoracolumbar epidural hematoma from the level of T10 to L2 with significant cord compression. The epidural mass was hyperintense on the T1W images (Figure 1).

**Fig. 2:**
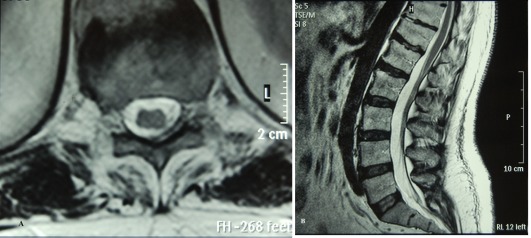
The lesion disappeared on the sagittal and axial sections on T1W images after a month

The patient was admitted to our department, an emergency decompression was recommended and operation preparing was started. But he refused surgical treatment. Therefore, he was managed conservatively with cessation of warfarin therapy and beginning of low-molecular-weight heparin therapy. He was not placed on intravenous or oral steroids due to his neurological complaint started 3 days ago. His complaint of weakness in lower extremities were gradually recovered in one week and he was mobilized. After one month, he regained full power and a control MRI was performed. MRI revealed the resolution of the thoracolumbar epidural hematoma totally (Figure 2).

## Discussion

Spontaneous spinal epidural hematoma is an uncommon cause of cord compression. The incidence of SSEH as estimated by Holtas et al. was 0.1 per 100,000 people and less than 1% of people with the condition, the spinal epidural space was occupied by lesions. The spontaneous development of spinal epidural hematomas is most frequent after the fourth or fifth decade. The male/female ratio was reported 1.5: 1.[1][2][5][6][7]

It has been reported to occur in all age groups. For instance, some pediatric cases of spinal subdural and epidural hematoma have been documented in the literature. They claimed that, aggressive surgical treatment should be delayed as long as possible in pediatric patients because of the spinal structure is still developing.[6][8]

The causative hematomas most frequently occur at the lower cervical and thoracolumbar spinal levels in adults, from C5 to T1 spinal levels in children.[7][9][10] Symptoms such as numbness, radicular paresthesis, progressive paraparesis appear within minutes to days.[3][11] Children often suffer from additional symptoms of irritability, and occasionally urinary retention.[12]

The etiology of SSEH is unknown, but predisposing factors such as increased venous pressure, hypertension, anticoagulant therapy for prosthetic cardiac valves, therapeutic thrombolysis for acute myocardial infarction, hemophilia B, factor XI deficiency, long term acetylsalicylic acid using as a platelet aggregation inhibitor, vascular malformation and pregnancy. However, the exact pathogenesis of the spinal epidural hematomas remains still obscure.[2][13][14][15]

Most authors have contended that, SSEH arise from epidural venous plexus in the spinal epidural space. Because of fluctuations in intrathoracic and intraabdominal pressures after exercise or other maneuvers, reversal of blood flow may induce rupture of a delicate vein in the valveless epidural plexus. Other researchers have proposed the spinal epidural arteries as a source of hemorrhage.[12][16]

MRI is the first choice diagnostic method for SSEH. If MRI is unavailable, CT scan should be obtained. In the differential diagnosis of other disease includes a spinal abscess, ischemia, transverse myelitis, acute herniated intervertebral disc and epidural tumor. MRI recognition of the blood products is the most important sign that distinguishes SSEH from other spinal extramedullary lesions. Spinal subdural hematoma was differentiated from spinal epidural hematoma. Spinal epidural hematoma has a more lentiform shape, and occasionally extends into the intervertebral foramina. On the contrary, spinal subdural hematoma has a crescent shape and traps the spinal cord or cauda equina.[8]

Our patient was admitted to our department with mild paraparesis and hypoesthesia. We decided to emergent surgical treatment and operation preparing was started. Also his warfarin therapy changed with low-molecular-weight heparin therapy. But the patient refused surgical treatment. Therefore, we decided to give him pain killers and strict bed rest with serial neurological examinations. After a week complaint of weakness in lower extremities, they recovered gradually. After three weeks, he was consulted with the Department of Cardiovascular Surgery and was managed with cessation of low-molecular-weight heparin therapy and beginning of warfarin therapy again. After a month, the patient was recovered completely. His MRI revealed the resolution of the thoracolumbar epidural hematoma totally.

So spontaneous spinal epidural hematoma was an uncommon cause of cord compression that commonly was considered as an indication for emergent surgical decompression. It should be considered in the differential diagnosis of the other conditions. In our case, the patient had mild paralysis and he was recovering gradually. So conservative treatment was recommended.
